# A review of malaria epidemiology and control in Papua New Guinea 1900 to 2021: Progress made and future directions

**DOI:** 10.3389/fepid.2022.980795

**Published:** 2022-10-31

**Authors:** Eimear Cleary, Manuel W. Hetzel, Archie C. A. Clements

**Affiliations:** ^1^Research School of Population Health, Australian National University, Canberra, ACT, Australia; ^2^WorldPop, School of Geography and Environmental Science, University of Southampton, Southampton, United Kingdom; ^3^Swiss Tropical and Public Health Institute, Allschwil, Switzerland; ^4^University of Basel, Basel, Switzerland; ^5^Curtin University, Perth, WA, Australia; ^6^Telethon Kids Institute, Perth, WA, Australia

**Keywords:** malaria, Papua New Guinea, malaria epidemiology, review, malaria control, Melanesia, Robert Koch

## Abstract

The research and control of malaria has a long history in Papua New Guinea, sometimes resulting in substantial changes to the distribution of infection and transmission dynamics in the country. There have been four major periods of malaria control in PNG, with the current control programme having commenced in 2004. Each previous control programme was successful in reducing malaria burden in the country, but multiple factors led to programme failures and eventual breakdown. A comprehensive review of the literature dating from 1900 to 2021 was undertaken to summarize control strategies, epidemiology, vector ecology and environmental drivers of malaria transmission in PNG. Evaluations of historical control programs reveal poor planning and communication, and difficulty in sustaining financial investment once malaria burden had decreased as common themes in the breakdown of previous programs. Success of current and future malaria control programs in PNG is contingent on adequate planning and management of control programs, effective communication and engagement with at-risk populations, and cohesive targeted approaches to sub-national and national control and elimination.

## Introduction

**Figure 1:**
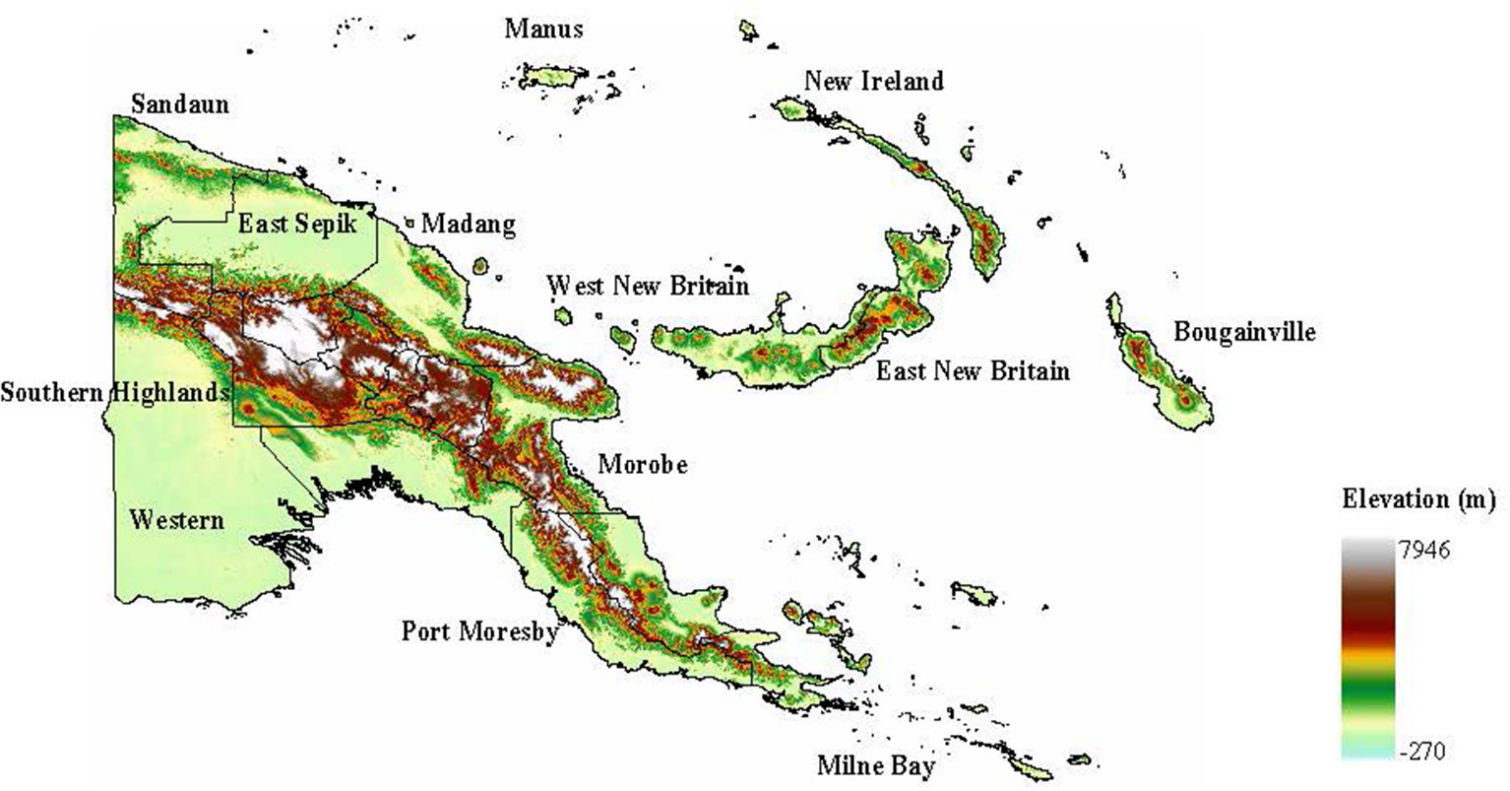
Global digital elevation map showing topographical diversity of PNG.

The population of Papua New Guinea (PNG) consists of over 8 million people spread across 22,000 villages and a small number of urban centers, including the fast growing capital city Port Moresby. Many of the villages, home to the majority of the population, are situated in rugged landscapes with difficult terrain resulting in many highly isolated communities and a population that is one of the most culturally, ethnically and linguistically diverse in the world (1, 2). PNG has a diverse geography and topography ranging from coastal lowlands, dense highlands rainforests, large wetland river basins and high mountain ranges (Figure 1) (3–5). This geographic diversity has contributed to extensive variation in human population distribution. Human settlement patterns in PNG have also historically been shaped by malaria transmission, with the population mostly living above 1,300 m, where temperatures are too low for sustained malaria transmission (4, 6), or below 600 m where the local population acquires partial immunity to infection (7–9). Heterogeneity in human behavior and population distribution, as well mosquito ecology, shaped by the diverse landscape has resulted in complex and heterogeneous patterns of malaria transmission and levels of endemicity (4, 10, 11).

A renewed commitment to malaria control in PNG in 2004 had resulted in a reduction in national prevalence to <1% in 2013/2014 (1, 6, 12, 13). However, historically, achievements in malaria control have been curtailed by funding constraints or humanitarian or natural disasters leading to a resurgence in malaria morbidity and mortality (14, 15). A leveling off in gains made globally in reducing malaria infection and mortality has been observed in recent years (16) and this too is a trend that has been observed in PNG, exacerbated to some degree by the COVID-19 crisis (17, 18). In 2020 ~80% of an estimated 1.7 million malaria cases in the World Health Organization (WHO) Western Pacific Region occurred in Papua New Guinea, with a case incidence of 164/1,000 population (18). PNG continues to have one of the highest levels of malaria prevalence outside Sub-Saharan Africa, with 94% of the population considered to be at risk (19).

A comprehensive review of published literature spanning over a century of malaria research in PNG was conducted to compile a historical summary of malaria epidemiology and control efforts that have shaped malaria transmission in PNG today. This review presents a summary of the successes and challenges faced by previous national malaria control programmes, from 1900 to present day, and examines the spatial distribution of malaria in PNG in response to the previous control efforts. Furthermore, this review also aims to elucidate reasons underpinning the breakdown and eventual failure of previous control programmes and subsequent rebound of malaria transmission, in order to inform current and future strategies for malaria control and elimination in PNG.

## The spatial epidemiology of malaria in PNG

Transmission patterns of malaria in PNG are diverse, complex and highly heterogeneous, exhibiting spatial variation at small scales (e.g., between and within villages) and between major geographic regions of the country (4, 11, 20, 21). For example, in 2008/2009, a country-wide malaria survey estimated a national *P. falciparum* prevalence of 12% but ranging from 0 to 49.7% across surveyed villages (10). Transmission ranges from perennial on the northern coast and most parts of the lowlands, to seasonal on the south coast with unstable transmission exhibited in the highlands in the center of the country (4, 22). The heterogeneity in transmission exhibited in PNG is attributed to a range of factors including varying use of long lasting insecticidal nets (LLINs) (11), human behavior (23), vector abundance and human biting rates, and climate (24, 25). Human genetic factors such as red blood cell polymorphisms which cluster in familial groups (4, 26, 27), differences in sporozoite and inoculation rates between villages, and response to control interventions also contribute to this heterogeneity in transmission (10, 28, 29). Individual, household and environmental factors can explain some of the differences in infection risk observed within villages (21).

Four human *Plasmodium* species are in circulation to varying degrees of endemicity in PNG: *P. falciparum, P. vivax, P. ovale*, and *P. malariae* (30). *P. falciparum* and *P. vivax* are the most common and clinically important species in the country with a spatial distribution that covers the entire country (10, 31). *P. falciparum* has been the predominant *Plasmodium* species across most of PNG (29) since the emergence of chloroquine resistant *P. falciparum* following cessation of a malaria control program in the early 1980s (31–33). However, a differential impact of control measures on *P. falciparum* and *P. vivax* following a large-scale distribution of LLINs in 2008/2009, led to an increase in the proportion of infections and cases due to *P. vivax* in some geographic locations in PNG (34–36).

Survey data collected in PNG in 2008/2009 observed that in some locations, the entomological inoculation rate with *P. vivax* was significantly higher than that of *P. falciparum* (34). Even prior to the scaling up of malaria control in 2008/2009, *P. vivax* endemicity in PNG was among the highest in the world (10). This observation may, however, have been as a result of recrudescent infections from *P. vivax* hypnozoites rather than recent vector-to-human infections (34, 37), or differences in impact of high coverage of LLINs to acquired immunity (38). Recent evidence from PNG concluded that, discounting *P. vivax* infection due to hypnozoite recrudescence, incidence of *P. falciparum* and *P. vivax* malaria was similar with comparable temporal and spatial distribution patterns (39). Although once highly prevalent in PNG (up to 13% in some areas), prevalence of *P. malariae* in PNG is comparatively low and *P. ovale* occurs only occasionally (4, 10).

The heterogeneity in the spatial distribution of malaria in PNG is driven, in part, by the diverse climate and environmental conditions in PNG with different climate conditions being more significant drivers of transmission in some locations than others (24). Temperature, rainfall and altitude are the most important environmental drivers of malaria transmission in PNG (4), although their relative impact differs to some degree between different geographical areas of the country (24, 40, 41). For example, temperature may be a significant driver of transmission in the lowlands or coastal areas, while in the highlands, altitude may be a better predictor of malaria prevalence (24, 30). Temperature is inversely related to mosquito infectivity, and changing temperature with increasing altitude is the main climatic determinant of malaria endemicity (3, 4, 42). Low temperatures and high altitude historically corresponded to low or unstable malaria transmission in the highland provinces (4, 30, 43). In areas of the south coast, malaria transmission is associated with rainfall and seasonality corresponds with the wet and dry seasons (4, 44).

The variation in spatial patterns of malaria transmission in PNG is also driven, in part, by the different vector dynamics and abundance across the country (25, 31). More than ten *Anopheles* species occupying different ecological niches are associated with *Plasmodium* transmission in PNG (31, 34). The five major human malaria vectors in PNG include *Anopheles farauti* sensu strictu (s.s.), *Anopheles hinesorum* (formerly *An*. *farauti* 2), *Anopheles farauti* 4 (the first three belonging to the *An. farauti* species complex), *Anopheles koliensis* and *Anopheles punctulatus* (31). Each species has a wide spatial distribution (31) coexisting to a certain extent, but with distinct ecological requirements and habitats (44). Marked variation in vector species distribution exists between the highlands and low-lying coastal areas (25, 31).

The variation in vector predominance in different geographical locations is associated mainly with ecological characteristics of adult mosquitoes and larval breeding habitats, driven by climate conditions and vegetation cover (31, 44). Natural or human made pools are the most common breeding sites used by all *Anopheles* malaria vector species (45), and availability and formation of these types of breeding site in PNG vary geographically and seasonally. For example, in the mountainous interior and highland provinces of PNG, steep slopes are less suitable for the formation of standing pools of water as rainfall runs off into small streams. In the dry season, when the water level of small streams is low, this run off may add abundance to small streams resulting in formation of suitable breeding habitats. In the wet season however, when stream water levels are higher, the extra abundance of water and heavy flow of the stream water may flush out breeding sites (44).

*Plasmodium* vectors in PNG have different host and biting preferences, with some being more anthropophilic and having higher inoculation rates because of higher abundance and biting rates of these species than others (28, 46). Preference for night time or day time biting also varies between *Anopheles* species in PNG. For example, *An. koliensis* and *An. punctulatus*. *An. koliensis* bite at night-time, both indoors and outdoors, and generally rest close to their breeding site after feeding rather than close to their next blood meal indoors (44, 47). *An. farauti* are night time and early evening biters and also occasionally bite during the day (22, 48). Although general biting patterns have been observed amongst specific *Anopheles* species in PNG, in recent years a change in biting behavior in PNG has been noted, most probably in response to control interventions (49, 50). Changes in biting and resting habits of vectors may influence susceptibility of vectors to IRS interventions and, as some *Anopheles* species are more resilient to control interventions than others, species composition is vulnerable to the control interventions and has been altered as a consequence in the past (22, 44, 49). For example indoor dichloro-diphenyl-trichloroethane (DDT) spraying pilot projects conducted during the 1950s were found to be effective against *An. punctulatus* and *An. koliensis*, but not against *An. farauti* (22, 51).

## History of malaria control in PNG

### Robert Koch's German Malaria Expedition in PNG: 1900

Malaria control efforts and malaria research in present-day PNG commenced with the arrival of microbiologist Robert Koch in the late 1800s (52–54). Some of the observations made by Koch in what was then known as German New Guinea formed the early basis of understanding of malaria epidemiology and immunology and transmission dynamics (3). Among these were the observations that malaria may be present in young children but not in adults, and in migratory populations from non-malarious areas of Europe, China and Melanesia rather than long term inhabitants of malaria endemic areas. These observations provided insight into the acquisition of immunity as a result of repeat exposure and infection; the specificity of immunity to distinct *Plasmodium* species rather than cross-species immunity; and occurrence of infection outbreaks as a result of migration of non-immune populations into endemic areas (3, 52–56). Koch investigations in PNG also identified *Anopheles* larval habitat as shallow sunlit pools with vegetation, fed by small streams, springs or flushes of rain water, and made early observations on the lifecycle of larval and adult *Anopheles* mosquitoes (56).

Malaria control strategies for PNG advocated by Koch were identification of all cases of malaria infection and treatment with quinine (56). Some of the earliest research into the chemotherapeutic application of quinine in the treatment of malaria was conducted by Robert Koch in the Madang district on the North coast of the island of New Guinea in 1900 (52, 53, 57, 58). Koch also emphasized the importance of identification and treatment of asymptomatic malaria infection (through repeated exposure or recrudescence) through active surveillance (56). Koch's assertions at the time were that national scale vector elimination was not possible, but that the elimination of malaria was possible through active surveillance and treatment of infection, as well as prevention of infection through use of mosquito nets, provided a sufficient number of doctors and supply of quinine was available (56, 59). Koch's malaria investigations in PNG ended with the departure of the German Malaria Expedition in September 1900 having “completed the tasks set by the commission”, but with Koch ultimately unsatisfied with the progress made toward malaria control (59).

### During and post-World War II: 1940s to 1950s

Prior to 1942 little progress in malaria control in PNG had made due to a paucity of financial and logistical resources (60). Interventions carried out between 1900s and 1940s were conducted on small subnational scales and focused on vector control efforts through drainage of breeding sites, elimination of larvae by use of the larvicidal fish*, Gambusia affinis*, spraying with DDT and infrastructure development around settled areas to prevent vector breeding (4, 7, 54). Beginning in 1942, during the Pacific War, malaria control efforts in PNG were again intensified and focused on vector control through use of mosquito nets, protective clothing and topical application of dimethyl-phthalate repellent, larviciding and draining of breeding grounds near campsites (7, 60–62).

Control efforts were concentrated predominantly on protecting troops, members of the local population involved in national defense and laborers working on coastal plantations and in tea and coffee plantations in highland regions (7, 57, 61). Highland transmission at the time resulted mainly from importation from the endemic lowlands and manifested as seasonal epidemics and outbreaks and was marked with substantial morbidity and mortality (43, 63, 64).

During this time period, DDT was considered the gold standard of insecticides but its use was limited to aerial spraying as the residual properties of DDT as an effective indoor insecticide had not yet been fully realized (60). In 1944 the first aerial spraying campaign with DDT was carried out by allied forces, but it carried an enormous financial cost and was of limited value (57, 60). During this time an additional, integrated community development malaria control strategy known as “bonification” was employed for malaria control, a 3-fold policy focusing promotion of wellbeing of the human population, water and agricultural land (60).

Between 1946 and 1948 infectious disease control interventions around Port Morseby included the burning off of vegetation in surrounding areas to expose habitats suitable for *Aedes aegypti* breeding (a major arbovirus vector). However, instead of controlling malaria this burning off of vegetation exacerbated transmission by facilitating formation of shallow sunlit ground pools, suitable as breeding grounds for local *Anopheles* species (23, 60). Incidentally, the cutting of grass around houses is still today considered a suitable practice for malaria control in PNG villages today (23). During this period of malaria control, mepacrine, an antimalarial drug related to chloroquine and mefloquine, was widely used as a preventative treatment for malaria, predominantly among allied forces during the second world war (65). Subsequent emergence of wide-scale resistance however resulted in discontinuation of its use (60).

From about 1950, as the burden of malaria was decreasing, efforts to sustain the bonification control program were beginning to wane, as was investment in the training of control specialists and malaria education. The intensified integrated intervention approach of aerial spraying with DDT, along with provision of protective clothing, mosquito nets, screens for houses, antimalarials and insect repellent to all at-risk populations which had been employed in the protection of troops during war time, were considered to be too expensive to be sustainable in “peace time administration” and were subsequently abandoned (60).

During the Second World War, infection prevention resources had been directed primarily toward Australian and American military personnel resulting in a high post-war burden of disease among the local population, with limited drug supply and resources to address it (60). To alleviate the high burden of disease, a community health led intervention program known as the “Aid Post Orderly Scheme” was initiated which involved the distribution of antimalarials supplied by the Department of Public Health, drainage of breeding grounds and larval control with *Gambusia* fish. Interventions were facilitated by aid post orderlies, with one person from each village being trained in malaria prevention and treatment methods (60).

Control programs in PNG implemented during this time had some success in halting epidemics and achieved a substantial reduction in prevalence across the country. However, problems with administration, training and discontent with the program, and reluctance of the general population to adhere to a disagreeable regimen of malaria prophylaxis with quinine, eventually contributed to a breakdown of the control program (4, 7). Program operations were also less effective on the islands due to poor coordination of activities during peaks and troughs in transmission and the change in vector biting behavior in response to spray interventions (7).

### WHO Global Malaria Eradication Program: 1950s to 1980s

From the 1950s to the 1980s, attempts were made to eliminate malaria in PNG using spraying with DDT and mass drug administration (MDA) with chloroquine (7, 29), coinciding with the WHO Global Malaria Eradication Program (66). The persistent nature of the insecticide on indoor surfaces meant that contact time with resting vectors was increased (60). DDT was therefore considered an effective malaria control intervention, as well as being safe and economically viable, and was used in well over half the households of PNG (7). The first pilot project on indoor residual house spraying (IRS) with DDT as a means of malaria control and elimination was undertaken in in 1957 in Maprik, Sepik province and the D'Entrecasteaux Islands in Milne Bay (7, 67). Initial studies showed a favorable response to DDT residual spraying and it was thought that malaria eradication was possible given the right resources.

By the early 1970s, the malaria control program primarily consisted of IRS with DDT, supplemented with MDA during malaria outbreaks, and environmental management in certain areas. Control measures covered ~50% of the population in 14 of the 19 administrative districts of PNG, and resulted in substantial, initial, reductions in malaria in many locations (7, 34). In the highlands, the spraying regimen operated once per year, and case detection was carried out by passive surveillance. In the islands and lowlands, where prevalence was higher, spraying was carried out twice a year and case detection was carried out by active surveillance. In the highland areas where infection prevalence was 5–10% prior to commencement of IRS, epidemics had ceased in all areas where the program was operational and parasite rates in many other areas had been greatly reduced (7, 68).

By 1974, after 10 years of integrated interventions, and in excess of 30 rounds of spraying in some parts of PNG, the control program was yielding poor results and was operational in only part of the country (57, 69, 70). Active monthly surveillance in the highlands was continued among a population of 20,000 people for a period of 18 months but was discontinued following detection of only three positive slides over the surveillance period. The low rates of infection detected during this period impacted the morale of surveillance teams and led to the conclusion that surveillance of this type was too resource intensive for an area of such low infection rates (7, 68). It was recommended that passive surveillance be carried out in areas where parasite rates are <2% and in hypoendemic areas in the highlands (7).

While the program had achieved some success in reduction of prevalence and interrupting transmission in the highlands (7), confidence in an eradication program was abating in favor of a more realistic goal of control (70, 71). At the time, malaria control using DDT was still effective although failure of some residual spraying rounds were being observed (7). No regular evaluation of vector susceptibility to DDT was carried out during the campaign, and emergence of insecticide resistance resulted in resurgence of local infection rates in some areas to a level higher than had existed prior to control program commencement (7, 35, 72). People became dissatisfied with IRS due to the alleged implication of DDT in the death of cats and fowl and associated proliferation of rats and bedbugs, which led to a general refusal to allow strangers to carry out spray operations in households (7, 69).

High or rising parasite rates existed in many parts where control measures had been operational for more than a decade, and despite availability, the number of people regularly taking antimalarials was low (7). Coupled with the fact that promises of eradication had been made when it was known that only control was possible, it is reasonable to assume that communities were becoming intervention fatigued and that trust in the malaria control program had been damaged (57, 60). The strategy of malaria eradication primarily through vector control with DDT spraying, with drug reinforcement when necessary, was not considered feasible and only considered possible if resources were “unlimited and enormous” (60).

Additional reasons for program failure were inconsistencies between different administrative areas in the running of the control program, caused by a lack of inter-district coordination and communication (7). The program was also hampered by inadequate water transport facilities, financial constraints, and too small a number of technical, administration and training staff. It was suggested that the training received by malariologists during the early 1970s placed too much of an emphasis on vector control through IRS with DDT and as a result of broader approaches employed by earlier programs being neglected (70). Health education was considered an essential precursor to any elimination program, but health educators were transferred to other positions when the elimination program started (60).

An eradication program was only believed to have been feasible had the following been achieved: nationwide coverage of the interventions, adequate planning, administration, operation and assessment, sustained financial backing, visible economic benefits, full government support, integration of the program with high quality national and local health services, and adequate health education (69, 70). Recommendations at the time were that effective disease control or eradication program in PNG should be simple and as cheap as possible consistent with reasonable efficiency” (60). The spraying program was ceased in the 1980s which, together with decentralization of malaria control administration and emergence of drug resistant *Plasmodium* parasites, resulted in a malaria resurgence with predominance of *P. falciparum*, across most parts of PNG in the 1990s (29, 33, 34).

### Papua New Guinea National Malaria control program and the Global Fund to Fight Aids, Tuberculosis and Malaria: 2004 to 2015

Between 2005 and 2009, the National Department of Health, in partnership with Rotarians Against Malaria, led the first free nation-wide distribution campaign of 2.4 million LLINs, resulting in 65% national ownership. The campaign was funded by a US$16 million grant awarded by the Global Fund to Fight AIDS, Tuberculosis and Malaria in 2004 (10, 12, 34). A further grant of US$102 Million, awarded in 2009, facilitated the continued distribution of an additional 2.5 million LLINs between 2009 and 2011 to all households in all provinces, resulting in an increase in country level ownership of at least one LLIN per household to 81.8% (1, 73). The national LLIN distribution campaign was followed by substantial reductions in human biting rates, malaria transmission and morbidity within 1 year (22, 34), and a reduction in malaria prevalence and incidence across PNG (6, 22, 25, 29).

**Figure 2:**
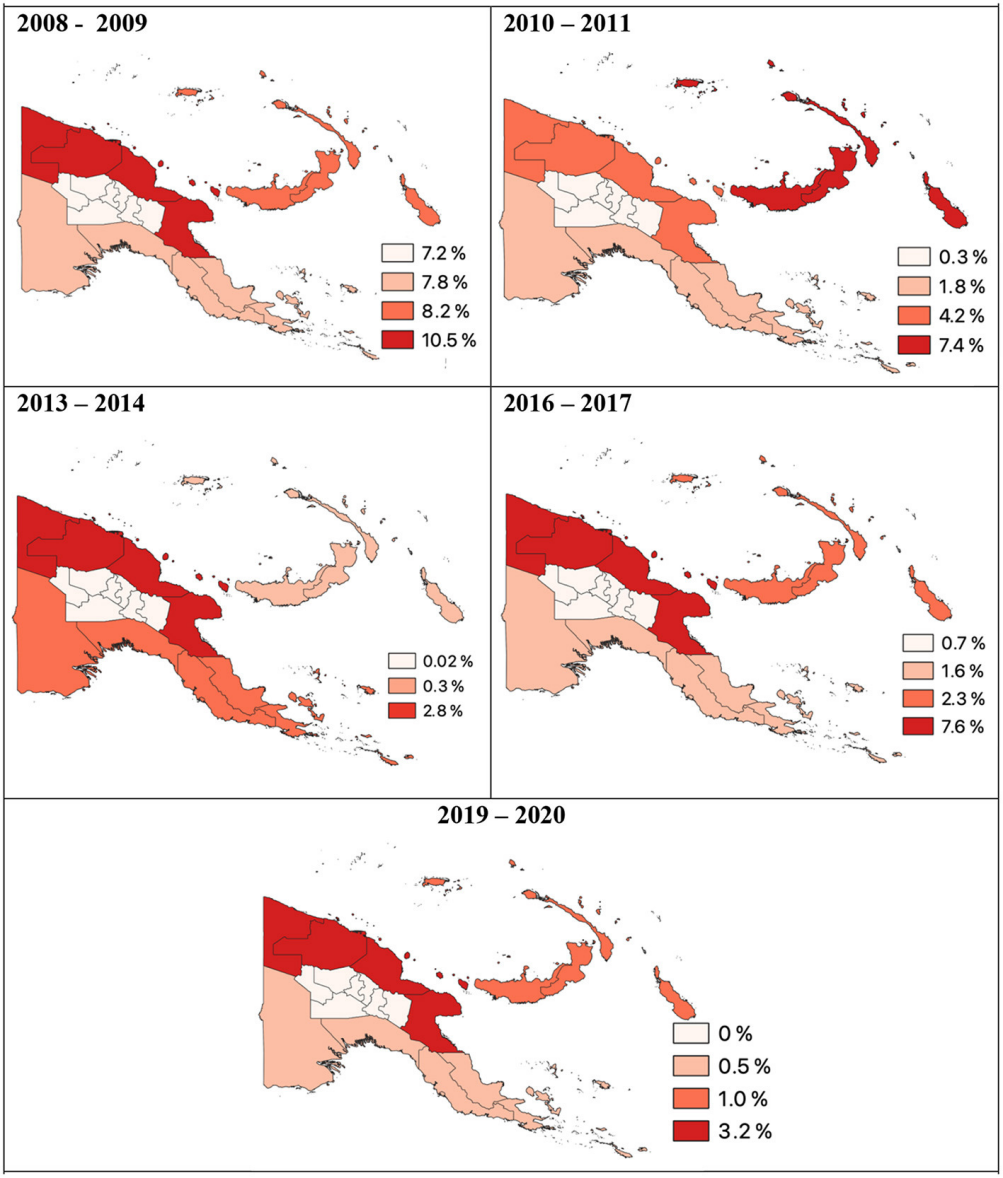
*P. falciparum* prevalence per region of Papua New Guinea 2008 to 2020. Data derived from Malaria Indicator Surveys (78).

Prior to commencement of renewed malaria control efforts financed by the Global Fund, prevalence of malaria infection in PNG varied widely, being <10% in some communities and more than 70% in others, with an average of between 35 and 45% and an estimated mortality rate of children under 10 attributed to malaria of 4–17% (25, 74–76). Results of a Malaria Indicator Survey (MIS) conducted in 2008–2009 estimated a prevalence range of 0–49.7%, with an average prevalence of 12% nationwide (Figure 2) (10). In the highland provinces little malaria control had been undertaken since the 1980s and by the early 2000s malaria prevalence in the highlands had rebounded to pre-control levels (63). Local epidemics were associated with substantial morbidity and high incidence of clinical infection (77).

**Figure 3:**
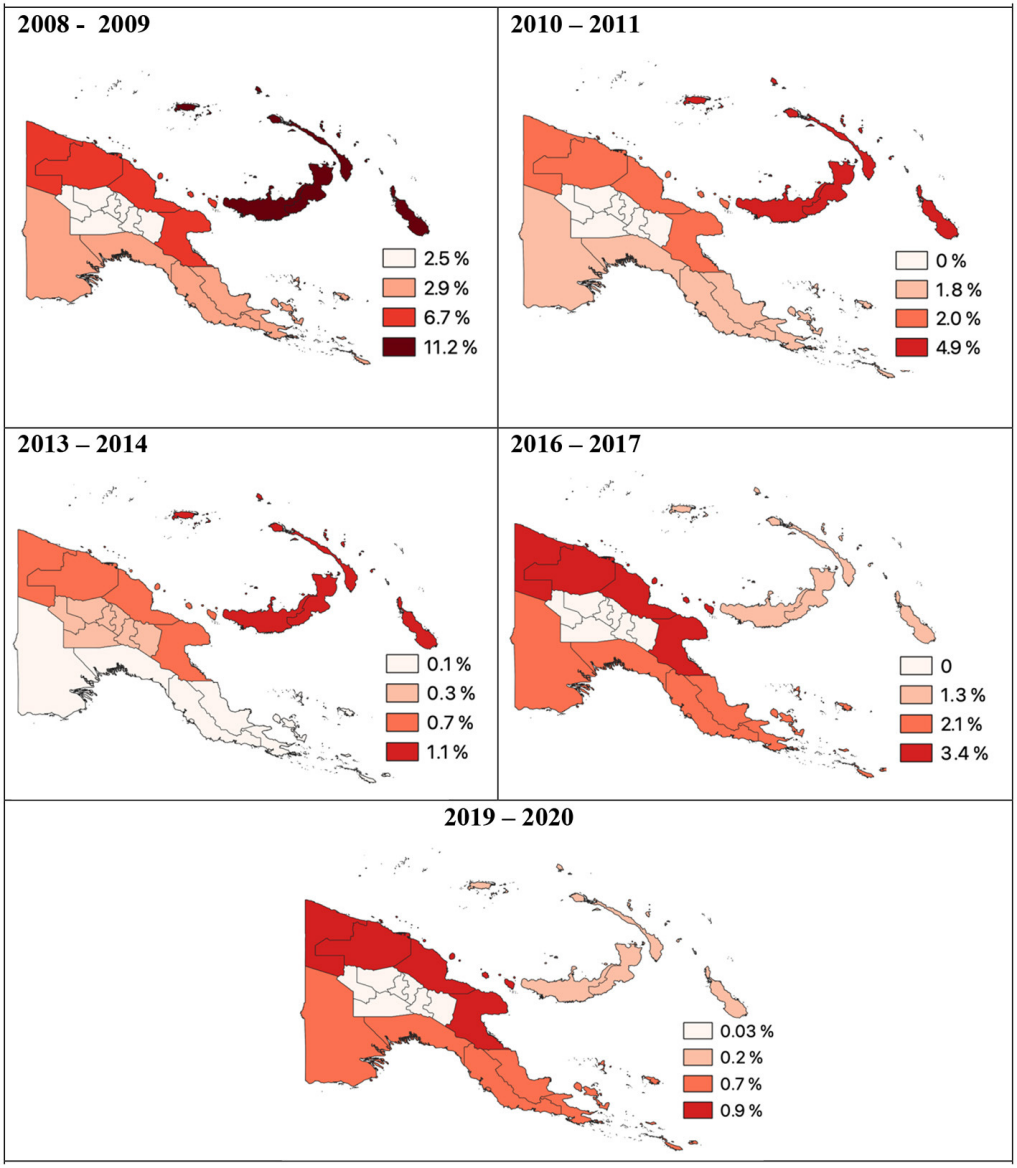
*P. vivax* prevalence per region of Papua New Guinea 2008 to 2020. Data derived from Malaria Indicator Surveys (78).

In 2013–2014 following the first two rounds of nation-wide LLIN distribution, national malaria prevalence in PNG had declined to <1% (78). In the lowlands, malaria prevalence decreased from 11.1% in 2008–2009 to 5.1% in 2010–2011 and then to 0.9% in 2013–2014 (6). Along the north coast of PNG prevalence of *P. falciparum* and *P. vivax* had decreased 12- and 6-fold, respectively (Figure 3) (79). Malaria prevalence had also decreased in the highlands as a consequence both of lower rates of infection in the highlands and less importation from the lowlands (6).

**Figure 4:**
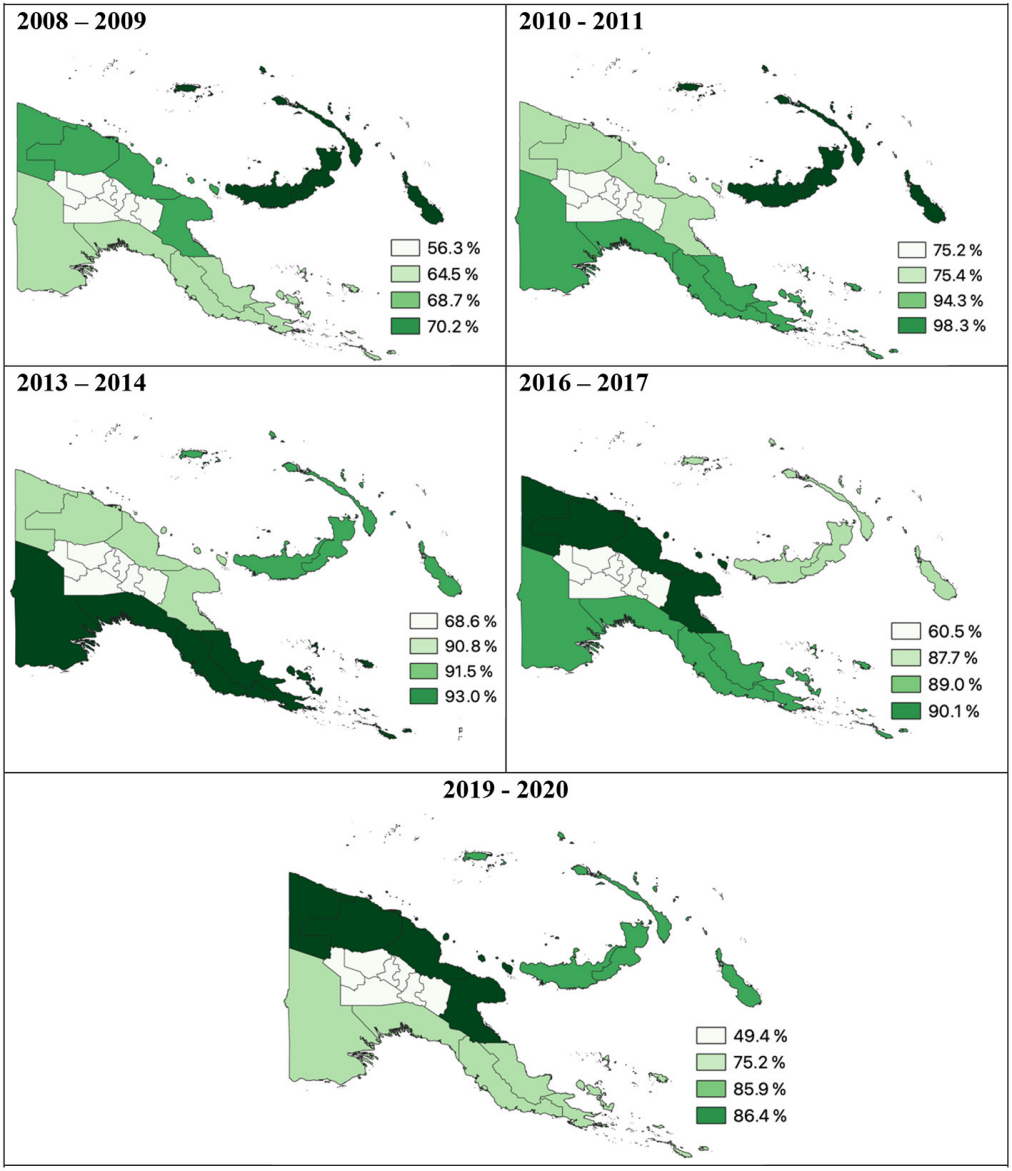
Percentage of households per region with at least one LLIN. Data derived from Malaria Indicator Surveys (78).

In 2009 following two rounds of LLIN distribution, reported average LLIN use was only 32.5% and varied across the country, ranging from 95–100% use in Madang and Western Province (Sausi and Balimo, respectively) to 21–69% in the Islands region (Arawa, Bougainville and Lemakot, New Ireland) (Figure 4) (29, 80). In a 2010–2011 survey, it was estimated that 81.8% of households owned at least one LLIN (73) and in the islands region, ownership increased from 29.3 to 98.3% (73). However, only an average of 41.3% of households in PNG (25.9% in Momase to 62.2% in the Islands region) owned a sufficient number of LLINs, defined as one LLIN per two people, even in areas with 88.8% household ownership (73).

As well as the free distribution of LLINs, the national malaria control program expanded to include RDTs or microscopy for diagnosis of febrile illness, behavior change campaigns and artemisinin-based combination therapy (ACT), specifically artemether-lumefantrine, for the treatment of clinical malaria episodes (29, 50, 81). Until 2002, diagnosis of malaria and treatment was administered on a presumptive basis (1, 4, 35) and fever cases were often treated as malaria (75, 82). New malaria treatment guidelines introduced in 2009 required a parasitological diagnosis of fever cases with RDT or light microscopy and treatment only in the case of a positive diagnosis (63, 83), a protocol which is now applied across the country (79, 84).

Since the introduction of the new treatment protocol here has been an increase in the percentage of health centers stocking RDTs from 17.5% in 2010 to 90.2% in 2012 and in the use of RDTs for diagnosis of febrile illness (84–86). In 2014, 85% of health centers surveyed were able to provide first-line treatment for uncomplicated malaria, and 42% of health facilities had first-line treatment available for severe malaria (84, 85, 87). However, survey evidence in 2012 found that a high proportion of fever cases (96.4% of fever patients) were being treated for malaria on a presumptive basis, even with a negative RDT test, which may eventually contribute to parasite resistance to anti-malarial drugs (88).

### Resurgence and reduction: 2015 to 2020

Despite nearly a decade of implementation of an intensive LLIN-based vector control program and steady LLIN ownership and usage, malaria continues to be transmitted at relatively high intensities in some villages (23, 25). Each subsequent round of LLIN distribution in PNG had resulted in an increase in self-reported LLIN use, and the MIS conducted in 2016–2017 found that 80.1% of all households owned at least one LLIN and 66.7% of the population had access to an LLIN (13, 29). However, after the third round of distribution, reductions in malaria prevalence were heterogeneous and the numbers of malaria cases in some locations were found to be increasing (29, 50).

The 2016–2017 MIS found an estimated 8.6 fold increase in prevalence of both *P. falciparum* and *P. vivax* compared to 2014 (13, 23, 50). National prevalence in areas below 1,600 m had rebounded to 7.1% and ranged from 8–16% in 5 out of 18 surveyed villages, and 0–5% in the remaining 13 villages (13). In the highlands (above 1,600 m), malaria infections were only detected in three villages and average prevalence was 0.9% (13) whereas in coastal villages in Madang Province *P. falciparum* prevalence ranged between 19.1–28.3% and *P. vivax* prevalence between 18.3–23.4% (13, 25).

Reasons for the rebound in transmission between 2013–2014 and 2016–2017 are not yet well-understood (89). Findings of the 2016–2017 MIS suggested that intervention coverage had plateaued and resurgence of malaria was likely to worsen unless vector control, diagnosis, treatment and behavior change campaigns are re-intensified (13). Although a decrease in mosquito abundance was observed in the first year after LLIN distribution, 2–3 years later, mosquito abundance had rebounded, accompanied by a change in the biting habits of *Plasmodium* vectors in PNG in response to vector control interventions and, in some areas, a change is species composition (22, 34). Early evening and morning outdoor biting among *Anopheles* species was reported following two rounds of LLIN distribution (49, 50), similar to previous observations following spray interventions during Koch's period of control intervention in PNG, and among *An. farauti* and *An. koliensis* species following an ITN distribution campaign in 1985 (22, 90). This shift in biting behavior following implementation of control interventions has also been observed elsewhere (91–93).

This earlier outdoor biting renders indoor vector control measures such as LLIN use and IRS less effective, increases exposure to infectious mosquito bites among people gathering or working outdoors in the early evening or morning, and may pose a substantial challenge to contemporary malaria control programs (23, 25, 47, 49). In addition to changes in biting behavior, recent evidence suggests a decrease in bioefficacy of LLINs distributed in PNG between 2012 and 2013 due to a change in the coating formulation of LLINs during manufacturing (94, 95). The durability of time period within which LLINs remain effective in PNG is also uncertain, and these factors may have a large impact on current and future malaria control efforts. Insecticide resistance, however has not yet been detected in PNG (96).

Following the resurgence of malaria in PNG between 2013–2014 and 2016–2017, the 2019–2020 MIS reported a reduction in national prevalence once again (89). The overall national prevalence of malaria was 2.1%, with a *P. falciparum* prevalence of 1.3 and 0.5% *P. vivax* prevalence (89). Reductions in prevalence were heterogenous however and have not returned to the low levels of national prevalence observed in 2013–2014 (0.9%) (78, 89). In the Highlands Region, malaria infections were detected in only two villages out of 40 villages attributed to importation of cases rather than local transmission (89). In West Sepik Province most surveyed villages had >10% prevalence and twelve villages with prevalence values >5% were found in all regions except the Highlands (89). In 2019–2020 national household ownership of at least one LLIN had declined to 69.3% with heterogenous ownership between low-lying areas (over 85% in Momase and the Islands Regions), and Southern and Highlands Regions (75.2 and 49.4%, respectively) (89).

### Lesson learned from the past and future directions for malaria control in PNG

While increases in funding and mass distribution campaigns in PNG have substantially increased access to LLINs, malaria diagnostics and treatment, barriers to effective interventions remain in PNG and use of these interventions may still need to be increased (23). The mountainous terrain, coupled with the high proportion of the population living in remote villages with poor infrastructure and access to basic services, makes distribution of interventions challenging (6, 42, 73). Often villages are only reachable by boat, air, or walking on foot for several days (1). Barriers to LLIN use also include perceptions of low malaria risk, a lack of knowledge regarding malaria transmission pathways and reluctance to use a LLIN in the heat, as well as insufficient availability (73, 80, 87, 89). Evidence also suggests a gender bias in LLIN use in PNG with adolescent and adult men less likely to use an LLIN than other household members (89).

Among the major challenges to achieving elimination of malaria in PNG will likely be factors such as changes in biting behavior of *Anopheles* vectors to daylight hours which will render LLINs less effective as preventative interventions. Dynamic vector species compositions in different geographic areas as well as heterogeneous biting behavior makes vector control using uniform interventions such as LLINs and IRS more difficult to achieve, and changes in transmission dynamics in response to control interventions should be evaluated by sustained surveillance (25). Insights from such investigations should be translated into vector control strategies that take heterogeneity in vector ecology into consideration with specific approaches targeting outdoor transmission where necessary (24, 29, 50).

Success of control programs in PNG is contingent on nationwide coverage of interventions directed by adequate planning and administration and sustained financial investment in the National Malaria Control program until, and after elimination, has been achieved (62, 69, 70, 97). Evaluations of previous control programs cite poor planning, a lack of cohesion in administration between different districts, insufficient resources and difficulty in sustaining financial investment and control efforts once malaria burden decreased as common themes in the breakdown of programs in the past (7, 69). High impact to high burden strategies including strategic targeting of interventions supported by fine-scale spatiotemporal risk mapping and continuing surveillance post sub-national elimination will be essential components of a coordinated national malaria response (24, 29, 50, 98). Genomic surveillance will also be key for identifying distinct transmission zones for targeting sub-national elimination strategies and preventing resurgence where elimination has been achieved, as well as detecting emergence of drug resistant *Plasmodium* parasites (63, 77, 99).

In countries where malaria-free certification has been achieved, risk of resurgence may be higher where complacency results in administrative and financial constraints and surveillance systems post elimination and availability of skilled personnel for diagnosis and treatment inadequate are insufficient (14, 100). Strategies that have been key to the success of countries achieving and maintaining zero malaria status include: robust surveillance systems and effective vector control, a well-trained workforce, prevention of importation through free diagnosis and treatment of malaria for international travelers (Cabo Verde, Bhutan); excellent health care systems that extend into remote areas (Algeria); early case detection through community-based health workers (Belize) and; a strict timeline for reporting detected malaria cases (China) (101).

Crucial to the success of future malaria control efforts in PNG will also be community engagement. Critical analysis of historical control efforts repeatedly cite poor communication during intervention delivery as key reasons for program failure. Control programs should consider local knowledge and human behaviors for targeting localized complementary interventions to accelerate efforts toward malaria elimination (23). Community led control strategies and engagement, as well as convenience of surveillance, treatment and preventative measures are also an essential aspect to the success of any infectious disease control campaign, as has been observed globally during COVID-19 pandemic. Grass roots movements such as Zero Malaria Starts With Me may be integral elements of community led strategies for subnational, and broader national, elimination campaigns (102).

Finally, additional challenges to successful outcomes of malaria control in PNG will access to well-functioning, staffed and resourced health care providers particularly among hard-to-reach populations (17). Distance to the nearest health facility has been reported as a factor in whether or not formal treatment is sought for suspected malaria, particularly in rural vs. urban and settlement areas (103). Any attempts at malaria elimination in PNG must include reaching marginalized groups living in rural, remote and border areas, which along with delivery of vector control interventions, is made difficult in PNG by the topological diversity of the country (15, 17, 29). Assessing risk of emergent artemisinin resistance and mosquito resistance to insecticides will be essential aspects to the successful control and elimination of malaria (104). Although all *Plasmodium* species in PNG remain susceptible to ACT, genetic screening has already detected presence of an ACT-resistant mutation in some *P. falciparum* isolates in PNG (105), and factors that reduce vector susceptibility to insecticides, including those used on LLINs, may eventually lead to malaria resurgence (25, 106).

## Conclusions

Following a sustained period of reduction in national malaria prevalence in PNG facilitated by mass LLIN distribution funded by successive grants from the Global Fund to Fight AIDS, Tuberculosis and Malaria and other donors, malaria transmission had resurged in PNG between 2013–2014 and 2016–2017. Although a decrease in national prevalence had been observed by 2019–2020, a rebound in malaria may again occur unless locally tailored effective preventative and curative interventions are implemented and sustained over time. Here we have presented a comprehensive overview of malaria epidemiology and a history of control programs in PNG, highlighting their successes and failures, in order to gain insights into strategies needed for success in current and future control and elimination efforts. Evaluations of previous control programs cite poor planning, a lack of cohesion in administration between different districts, insufficient resources and poor communication during intervention delivery as key reasons for program failure. Critical to the success of malaria control and elimination in PNG will be sustained investment of finances and resources, community engagement and locally led control strategies, and continued surveillance to prevent rebound of transmission once the burden of malaria has been diminished.

## Author contributions

EC and AC conceived the theme of this literature review. MH recommended substantial proportion of published the research literature reviewed for this paper. EC conducted a review of the published work, wrote the manuscript, and edited the final version of the manuscript. AC and MH provided comments. All authors contributed to the article and approved the submitted version.

## Funding

This study was funded through the Australian National University Postgraduate Award and the Global Fund to Fight AIDS, Tuberculosis and Malaria.

## Conflict of interest

The authors declare that the research was conducted in the absence of any commercial or financial relationships that could be construed as a potential conflict of interest.

## Publisher's note

All claims expressed in this article are solely those of the authors and do not necessarily represent those of their affiliated organizations, or those of the publisher, the editors and the reviewers. Any product that may be evaluated in this article, or claim that may be made by its manufacturer, is not guaranteed or endorsed by the publisher.
